# Clinical Practice Guidelines and Evidence for the Efficacy of Traditional Japanese Herbal Medicine (Kampo) in Treating Geriatric Patients

**DOI:** 10.3389/fnut.2018.00066

**Published:** 2018-07-23

**Authors:** Shin Takayama, Ryutaro Arita, Akiko Kikuchi, Minoru Ohsawa, Soichiro Kaneko, Tadashi Ishii

**Affiliations:** Department of Education and Support for Regional Medicine, Department of Kampo Medicine, Tohoku University Hospital, Sendai, Japan

**Keywords:** review, herbal medicine, kampo medicine, guidelines, geriatrics, elderly

## Abstract

Frailty is defined as a state of increased vulnerability to poor resolution of homeostasis following stress, which increases the risk of adverse outcomes such as falls, delirium, and disability in the elderly. Recently in Japan, clinical practice guidelines (CPG) have recommended kampo treatment. We conducted a search for reports on Japanese CPG and kampo medicine in the treatment of symptoms in the elderly. The search was performed using the databases PubMed, Ichushi Web, J-Stage, Japan Medical Publishers Association, Medical Information Network Distribution Service, and CPG containing kampo products in Japan; reports from January 1st, 2012 to October 31st, 2017 were reviewed. Over the past 5 years, nine CPGs have recommended kampo treatment based on the evidence for improvement in skin symptoms, cough, gastro-intestinal dysfunction, urinary dysfunction, and dementia. Treatments with kampo medicine are performed depending on the coexistence of manifestations based on the original kampo concept, i.e., cognitive dysfunction and dementia with sarcopenia showing urinary disorder. Each kampo formula includes multiple crude drugs that have several pharmacological functions; these drugs include alkaloids, glycosides, and polysaccharides. Thus, kampo formula has an effect on multiple organs and coordinates the relationship between the brain, endocrine system, immune system, and skeletal muscles. Kampo treatment can be considered as supporting holistic medicine in elderly individuals with frailty.

## Introduction

**Figure 1:**
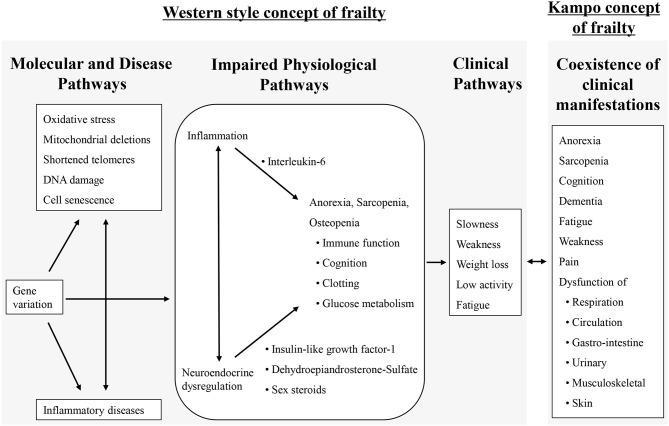
An overview of the hypothesized molecular, physiological, and clinical pathways involved in frailty and the kampo concept of frailty [adapted from (2, 5)].

Frailty is defined as a state of increased vulnerability to poor resolution of homeostasis following stress, which increases the risk of adverse outcomes such as falls, delirium, and disability in the elderly (1–3). Frailty involves disorders in multiple inter-related physiological systems, including the brain, the endocrine system, the immune system, and the skeletal muscle, resulting in weight loss, exhaustion, low energy expenditure, slow gait speed, and weak grip strength (4). The left side of Figure 1 shows an overview of the hypothesized molecular, physiological, and clinical pathways involved in the pathogenesis of frailty (5). In addition to exercise intervention combined with nutrition, multifunctional pharmacological agents are probably required for preventing frailty.

In the super aged society in Japan, the percentage of the elderly population (aged over 65 years) is expected to exceed 30% in 2025 and reach 39.9% in 2060 (6). Frailty is one of the reasons for need of nursing care in the elderly, thus the medical, social, and economical correspondence has been a research focus. Traditional Japanese medicine (kampo) has been applied for the treatment of comorbid symptoms or disorder for the past 1500 years. Kampo medicine includes multiple crude drugs that have several pharmacological functions including alkaloids, glycoside, and polysaccharide (7). Thus, kampo medicine has an effect on multiple organs and coordinates the relationship between the brain, endocrine system, immune system, skeletal muscles, and the patients' emotional condition. The right side of the Figure 1 shows the kampo concept for frailty. We believe that concomitant use with western medicine and kampo medicine can contribute to management of patients with frailty. Evidence based on clinical studies has grown in the past two decades; and recently, in Japan, clinical practice guidelines (CPG) for symptoms in the elderly gave some recommendations for use of kampo medicine.

In this report, we review CPG regarding kampo and the evidence of the efficacy of kampo medicine in the treatment of the elderly. We also discuss the characteristics of the kampo concept.

## Materials and methods

### Literature search

We conducted data base searches for Japanese CPG and kampo medicine for the symptoms in elderlies in PubMed, Ichushi Web, J-Stage, Japan Medical Publishers Association, Medical Information Network Distribution Service, and Clinical Practice Guideline Containing Kampo products. The search was restricted to CPGs published between January 1st 2012 to October 31st 2017. We used the following search strategy: 1. CPG; 2. Kampo medicine; 3. Herbal medicine; 4. #1–3 in Japanese; 5. #1 OR #2 AND #3 OR; and 6. #4 OR #5.

### Selection criteria

We included research articles published in English or Japanese that were related to CPG using kampo medicine. CPGs that mentioned the recommendation strength with quality of evidence and studies that included elderly subjects were selected. Physical therapy, massage therapy, acupuncture, and acupuncture-related techniques were excluded.

### Data extraction

Eligible articles were categorized by two independent researchers (ST and SK). Specifically, information from the articles or CPGs was extracted and tabulated, and eligible CPG were classified according to the name of CPG, symptoms or diseases, clinical question, mention about quality of evidence, and recommendation strength.

## Results

### General aspects

**Figure 2:**
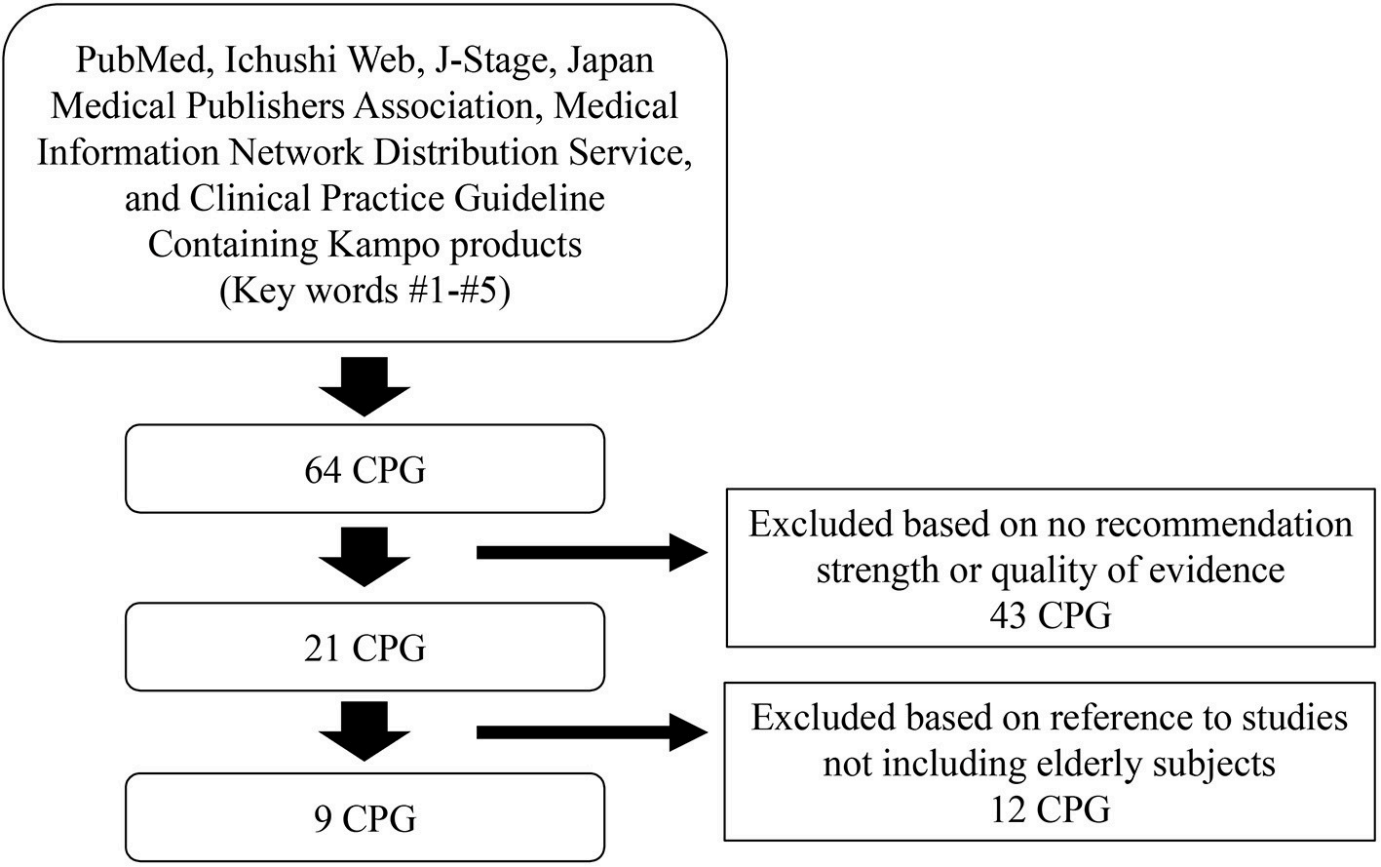
Flowchart of the review process. CPG, clinical practice guideline.

The review process flowchart is shown in Figure 2; and the eligible PCGs are shown in Table 1. Over the past 5 years, the following eligible nine CPGs have given some recommendations for kampo treatment based on the evidence for skin symptoms, allergy, cough, gastro-intestinal dysfunction, urinary dysfunction, and dementia: Clinical Practice Guideline for the Pruritus cutaneus universalis (8), Practical Guideline for the Management of Allergic Rhinitis in Japan (9), The Japanese Respiratory Society guidelines for management of cough (10), Evidence-based Clinical Practice Guidelines for GERD (11), Evidence-based Clinical Practice Guidelines for Functional Dyspepsia (12), Evidence-based Clinical Practice Guidelines for Irritable Bowel Syndrome (13), Evidence-based clinical practice guidelines for chronic constipation (14), Clinical guidelines for overactive bladder syndrome (15), and Practice Guideline for Dementia (16).

**Table 1:** Overview of CPGs included in the study.

**Clinical practice guideline**	**Clinical question**	**Symptom**	**Kampo medicine**	**Evidence (number of RCT including elderly subjects)**	**Recommendation**	**Publication year**	**Reference**
Clinical Practice Guideline for the Pruritus cutaneus universalis	Is kampo medicine effective for pruritus?	Skin pruritus, Senile pruritus	tokiinshi, orengedokuto, goshajinkigan, hachimijiogan, rokumigan	At least one RCT (5)	Permit	2012	(7)
Practical Guideline for the Management of Allergic Rhinitis in Japan	What are the indications for effective kampo medicine treatment in patients with allergic rhinitis?	Allergic Rhinitis	shoseiryuto	Double-blind RCT (1)	Recommend	2015	(8)
The Japanese Respiratory Society guidelines for management of cough	What is a non-specific therapeutic agent for dry cough?	Dry cough	bakumondoto	At least one RCT (1)	Recommend	2012	(9)
	What is a non-specific therapeutic agent for moist cough?	Moist cough	shoseiryuto	At least one RCT (1)	Recommend		
Evidence-based Clinical Practice Guidelines for GERD 2015 (2nd Edition)	Identification of drug with additional gastro-intestinal regulation effect of or kampo medicine combined with proton pump inhibitor.	GERD (addition to PPI, refractory to PPI)	rikkunshito	Low quality of evidence (1)	Low recommendation	2015	(10)
Evidence-based Clinical Practice Guidelines for Functional Dyspepsia	Is kampo medicine effective for Functional Dyspepsia?	Functional Dyspepsia	rikkunshito, (hangekobokuto)	High quality of evidence (2)	Low recommendation	2014	(11)
Evidence-based Clinical Practice Guidelines for Irritable Bowel Syndrome	Is kampo medicine effective for Irritable Bowel Syndrome?	Irritable Bowel Syndrome	keishikashakuyakuto	Low quality of evidence (1)	Low recommendation	2014	(12)
Evidence-based clinical practice guidelines for chronic constipation 2017	Is kampo medicine effective for chronic constipation?	Chronic constipation	daiokanzoto, daikenchuto	Low quality of evidence (2)	Low recommendation	2017	(13)
Clinical guidelines for overactive bladder syndrome		Overactive bladder	goshajinkigan	Small scale RCT (1)	Recommend	2015	(14)
Practice Guideline for Dementia 2017	Patient indications for effective non-medication or medication for agitation.	Agitation	yokukansan	Low (1)	Suggest	2017	(15)
	Patient indications for effective non-medication or medication for hallucinations and delusions.	Hallucinations, delusions	yokukansan	Low (0)	Suggest		
	How do you identify correspondence to dysphagia (including prevention for pneumonia)?	Correspondence to dysphagia (including prevention for pneumonia)	hangekobokuto	Very low (1)	Suggest		
	How do you respond in case of edema? (side effect)	Edema	yokukansan	Low (0)	Suggest		
	Is there treatment for behavioral and psychological symptoms of dementia, and for the sleep behavior disorder associated with Lewy body disease, and the rem period?	Behavioral and psychological symptoms of dementia and the sleep behavior disorder symptom of patients with Lewy body disease	yokukansan	Low (0)	Suggest		

### Randomized clinical trial involving elderly patients with each symptom introduced in the clinical practice guidelines

#### Skin symptoms

CPG for pruritus gave some recommendations (permission) for pruritus or dry skin based on some RCTs. Five RCTs for skin symptom were selected in the CPG focused on the elderly. Ohkawara et al. reported the efficacy and safety of orengedokuto or goshajinkigan for the symptom in senile pruritus (17). Orengedokuto (Huang-Lian-Jie-Du-Tang) (18) or goshajinkigan (Niu-Che-Shen-Qi-Wan) (18) improved the subjective and objective symptom with no significant difference in the efficacy and safety as compared to Clemastine Fumarate that is potent and selective histamine H1 receptor antagonist. Iida et al. reported the efficacy of tokiinshi (Dang-Gui-Yin-Zi) (18) for moisture holding ability evaluated with a measurement device for the water content of the skin corneum in patients with senile pruritus (19). Tokiinshi significantly improved dry skin compared with no treatment. Ishioka et al. reported the efficacy of hachimijiogan (Ba-Wei-Di-Huang-Wan) (18) for the subjective symptom in patients with senile pruritus (20). Hachimijiogan improved the subjective symptom with no significant difference in efficacy compared to Ketotifen Fumarate that is selective histamine H1 receptor antagonist. In addition, Ishioka reported no significant difference in efficacy between hachimijiogan and rokumigan (Liu-Wei-Wan) (18) for treatment of subjective symptom in patients with senile pruritus (21). Ohkuma et al. reported that orengedokuto with tokiinshi, orengedokuto alone, or tokiinshi alone significantly improved the itchy symptom with no significant difference in efficacy compared with anti-histamine (22).

#### Allergy symptoms

CPG for nasal allergy recommended use of kampo medicine for perennial nasal allergy with a referred RCT. Baba et al. reported the efficacy and safety of shoseiryuto (Xiao-Qing-Long-Tang) (18) for perennial allergic rhinitis in a double-blind RCT including elderly subjects (23). The shoseiryuto group showed more moderate to high improvement than that of the placebo group, with statistical significance. Mild side effects were detected in both groups with no significant group-wise difference.

#### Cough symptoms

CPG for cough recommended use of kampo medicine for cough in the elderly with two referred RCTs focused on the elderly. Irifune et al. reported the efficacy of bakumondoto (Mai-Men-Dong-Tang) (18) for post-infectious prolonged cough in elderly patients (24). As compared to procaterol hydrochloride, bakumondoto with procaterol hydrochloride was more effective in treating post-infectious prolonged cough. Mukaida et al. reported the efficacy of bakumondoto for cough in elderly patients diagnosed with chronic obstructive pulmonary disease (25). Additional bakumondoto treatment was more effective than regular medication in suppressing the severity of cough.

#### Gastro-intestinal symptoms

CPG for Gastro Esophageal Reflux Disease (GERD), Functional Dyspepsia (FD), Irritable Bowel Syndrome (IBS), and chronic constipation gave weak recommendation for use of kampo medicine for these conditions with referred RCT. In a double-blind RCT including elderly patients, Tominaga et al. reported the efficacy of rikkunshito (Liu-Jun-Zi-Tang) (18) for patients with GERD who were refractory to treatment with proton-pump inhibitor (PPI) (26). Rikkunshito combined with PPI significantly decreased the frequency scale of the GERD symptoms' score, similar to the decrease seen on treatment with a double dose of PPI. Subgroup analysis showed that the patients of male sex or low body mass index showed more improvement compared with the other subgroups.

Harasawa et al. reported that the regular dose of rikkunshito significantly improved dysmotility-like dyspepsia than the low dose of rikkunshito in a double-blind RCT including elderly subjects (27). Arai et al. reported that there was a significant improvement in dyspeptic symptoms in patients treated with either rikkunshito or domperidone that is peripheral D2-like receptor antagonist, based on the Gastrointestinal Symptom Rating Scale score (28). The improvements of reflux and indigestion symptoms in patients treated with rikkunshito showed good correlations with the increased levels of acylated ghrelin.

Sasaki et al. reported the efficacy and safety of keishikasyakuyakuto (Gui-Zhi-Jia-Shao-Yao-Tang) (18) for functional abdominal pain of irritable bowel syndrome in a double-blind RCT including elderly subjects (29). Abdominal pain in irritable bowel syndrome with diarrhea significantly decreased after keishikasyakuyakuto administration, and the effect was superior to that of a placebo. Adverse effects of keishikasyakuyakuto were not significantly different from the placebo, suggesting the safety of keishikasyakuyakuto.

Miyoshi et al. reported the efficacy and safety of daiokanzoto (Da-Huang-Gan-Cao-Tang) (18) for constipation in a double-blind RCT including elderly subjects (30). Daiokanzoto was significantly effective for constipation compared to the placebo. There were no significant differences in safety between daiokanzoto and the placebo.

Horiuchi et al. reported the effect of daikenchuto (Da-Jian-Zhong-Tang) (18) in patients with chronic constipation in an RCT including elderly subjects (31). The addition of daikenchuto to sennoside resulted in significant improvement in bloating and abdominal pain and significant decrease in the gas volume score.

#### Urinary symptoms

CPG for overactive bladder syndrome gave weak recommendation for use of kampo medicine with a referred RCT (32). In an RCT including elderly patients, Nishizawa et al. reported the efficacy of goshajinkigan for patients with over active bladder compared with propiverine hydrochloride. During the 1-year observation period, propiverine hydrochloride significantly improved the symptom of over active bladder in the first month, however, goshajinkigan significantly improved the symptom of over active bladder after 2 months. The incidence of adverse event related to treatment with goshajinkigan was lower than that with propiverine hydrochloride.

#### Dementia and related symptoms

CPG for dementia and related symptom gave weak recommendation for use of kampo medicine with two referred RCTs. Mizukami et al. reported the effectiveness and safety of yokukansan (Yi-Gan-San) (18) for the treatment of behavioral and psychological symptoms of dementia (BPSD) in patients with dementia associated with Alzheimer's disease or Lewy bodies (33). The mean total of the Neuropsychiatric Inventory score used to evaluate BPSD were significantly improved by the additional yokukansan administration. Subscale analysis showed significant improvements in delusions, hallucinations, agitation/aggression, depression, anxiety, and irritability/lability. There were no serious adverse reactions. Iwasaki et al. examined the effect of hangekobokuto (Ban-Xia-Hou-Pu-Tang) (18) on swallowing reflex among the elderly (34). Compared to the placebo, hangekobokuto significantly improved time from the water injection to the onset of swallowing. Furthermore, substance P in the saliva of patients administered with hangekobokuto significantly increased. Based on the result, Iwasaki et al. reported that treatment with hangekobokuto reduced the risk of pneumonia and pneumonia-related mortality in elderly patients with dementia in an RCT with 12-month patients' follow-up (35).

### Adverse effects

Adverse effects were not searched by the above steps. Additional hand searching for adverse effects showed that four CPG indicated the side effect of kampo medicine and introduce caution for using kampo medicine.

Guidelines for the management of hypertension 2014 (36) introduces the side effect of kampo medicine, western medicinal drugs, or health supplement that includes Glycyrrhiza. Glycyrrhiza includes glycyrrhizin which has potential to induce pseudo aldosteronism. If increase of blood pressure and/or hypokalemia are confirmed, withdrawing of suspicious products should be considered. In case it is difficult to stop use of the products, aldosterone antagonist can be prescribed.

Clinical Practice Guideline for the Management of Upper Tract Urothelial Carcinoma (37) introduces the side effect of crude drugs including aristolochic acid. Some studies indicated that aristolochic acid was associated with nephropathy, uropathy, or urothelial cancer. In Japan, crude drugs approved by the Ministry of Health, Labor and Welfare do not include aristolochic acid, but patients can use imported drugs.

Consensus statement for the diagnosis and treatment of drug-induced lung injuries (38) introduced the side effect of kampo medicine, especially shosaikoto (Xiao-Chai-Hu-Tang) (18), which can induce interstitial pneumonia in rare cases. The prevalence of shosaikoto-induced interstitial pneumonia is reported as under 0.1%, however, national survey reported the mortality rate of 10 in 100 cases of interstitial pneumonia induced by shosaikoto. If drug-induced interstitial pneumonia is expected, use of the suspected drug should be discontinued.

The guideline Japanese Society of Laboratory Medicine 2015 (39) introduced drug-induced edema, hypertension, and liver dysfunction. Comments for edema and hypertension induced by Glycyrrhiza were similar with prior comments in Guidelines for the management of hypertension 2014. Kampo medicine is one of the drugs that induce liver injury or hepatitis, therefore side effect correspondence is considered important.

## Discussion

### Interpretation of results

Nine CPG gave some recommendations for total 13 kampo medicines in treatment of the symptom in the elderly. Each kampo medicine includes multiple crude drugs which have several pharmacological functions; thus, kampo medicine affects multiple organs and coordinates the interrelation between the brain, endocrine system, immune system, and skeletal muscle in elderly patients.

### Characteristics of kampo medicine introduced in CPGs

Table 2 show the over view of 13 kampo medicines introduced in CPG, its indications and components referred from STORK (40), and its characteristics in kampo theory. Kampo medicine have multiple effect on organs result in improving symptom.

**Table 2:** Over view of 13 kampo medicines introduced in CPG, its indications and components referred from STORK (40), and its characteristics in kampo theory.

		**Kampo medicine**												
		**orengedokuto**	**goshajinkigan**	**tokiinshi**	**hachimijiogan**	**rokumigan**	**shoseiryuto**	**bakumondoto**	**rikkunshito**	**keishikashakuyakuto**	**daiokanzoto**	**daikenchuto**	**yokukansan**	**hangekobokuto**
Clinical manifestation of frailty according to the traditional concept	Anorexia							◦	◦			◦	◦	
	Sarcopenia		◦		◦	◦								
	Cognition		◦		◦	◦								
	Dementia	◦											◦	
	Fatigue								◦					◦
	Weakness		◦		◦	◦			◦					
	Pain		◦		◦					◦		◦		
	Dysfunction of													
	Respiration						◦	◦						◦
	Circulation		◦	◦	◦	◦								
	Gastro-intestine							◦	◦	◦	◦	◦		◦
	Urinary		◦		◦	◦								
	Musculoskeletal		◦		◦	◦								
	Skin	◦	◦	◦	◦	◦								
Indications		The relief of the following symptoms of those patients who have ruddy face with comparatively strong constitution, a touch of hot flushes, and a tendency to irritability: nose bleeding, hypertension, insomnia, neurosis, gastritis, alcoholic hangover, climacteric disturbance and automatic imbalance syndrome peculiar to women resembling climacteric disturbance, dizziness, palpitation, eczema or dermatitis and pruritus cutaneous	The relief of the following symptoms of those patients with decreased urine volume or polyuria sometimes having dry mouth who are easily fatigued and easily feel cold in the extremities: Leg pain, low back pain, numbness, blurred vision in old patients, pruritus, dysuria, frequent urination and edema	The relief of the following symptoms of those patients with oversensitivity to cold: Chronic eczema (with little exudation) and itching	The relief of the following symptoms of those patients with severe fatigue or malaise, decreased urinary output or increased urinary frequency, dry mouth, and alternate cold and hot feeling in the extremities: Nephritis, diabetes mellitus, impotence, sciatica, low back pain, beriberi, cystorrhea, prostatic hypertrophy and hypertension	The relief of the following symptoms of those patients with decreased urine volume or polyuria sometimes having dry mouth who are easily fatigued: Dysuria, frequent urination, edema and pruritus	Watery sputum, watery nasal discharge, nasal obstruction, sneezing, stridor, coughing, lacrimation in the following diseases: Bronchitis, bronchial asthma, rhinitis, allergic rhinitis, allergic conjunctivitis, and common cold	The relief of the following symptoms: Coughing with a hard, obstructive sputum, bronchitis, and bronchial asthma	The relief of the following symptoms of those patients with weak stomach, loss of appetite and full stomach pit, and those who are easily fatigued, anemic and likely to have cold limbs: Gastritis, gastric atony, gastroptosis, maldigestion, anorexia, gastric pain, vomiting	The relief of the following symptoms of those patients with abdominal distension: Tenesmus alvi and abdominal pain	Constipation	The relief of abdominal cold feeling and pain accompanied by abdominal flatulence	The relief of the following symptoms of those patients with delicate constitution and nervousness: Neurosis, insomnia, night cry in children, and peevishness in children	The relief of the following symptoms of those patients who have depressed feelings and a feeling of foreign body in the throat and oesophagus and who sometimes have palpitation, dizziness, nausea, etc.: Anxiety neurosis, nervous gastritis, hyperemesis gravidarum, coughing, hoarseness, nervous oesophageal stricture, and insomnia
Components of kampo medicine		JP Scutellaria Root	JP Rehmannia Root	JP Japanese Angelica Root	JP Rehmannia Root	JP Rehmannia Root	JP Pinellia Tuber	JP Ophiopogon Tuber	JP Atractylodes Lancea Rhizome	JP Peony Root	JP Rhubarb	JP Processed Ginger	JP Atractylodes Lancea Rhizome	JP Pinellia Tuber
		JP Coptis Rhizome	JP Achyranthes Root	JP Rehmannia Root	JP Cornus Fruit	JP Cornus Fruit	JP Processed Ginger	JP Brown Rice	JP Ginseng	JP Cinnamon Bark	JP Glycyrrhiza	JP Ginseng	JP Poria Sclerotium	JP Poria Sclerotium
		JP Gardenia Fruit	JP Cornus Fruit	JP Tribulus Fruit	JP Dioscorea Rhizome	JP Dioscorea Rhizome	JP Glycyrrhiza	JP Pinellia Tuber	JP Pinellia Tuber	JP Jujube		JP Zanthoxylum Fruit	JP Cnidium Rhizome	JP Magnolia Bark
		JP Phellodendron Bark	JP Dioscorea Rhizome	JP Peony Root	JP Alisma Rhizome	JP Alisma Rhizome	JP Cinnamon Bark	JP Jujube	JP Poria Sclerotium	JP Glycyrrhiz		JP Koi	JP Uncaria Hook	JP Perilla Herb
			JP Plantago Seed	JP Cnidium Rhizome	JP Poria Sclerotium	JP Poria Sclerotium	JP Schisandra Fruit	JP Glycyrrhiza	JP Jujube	JP Ginger			JP Japanese Angelica Root	JP Ginger
			JP Alisma Tuber	JP Saposhnikovia Root and Rhizome	P Moutan Bark	JP Moutan Bark	JP Asiasarum Root	JP Ginseng	JP Citrus Unshiu Peel				JP Bupleurum Root	
			JP Poria Sclerotium	JP Polygonum Root	JP Cinnamon Bark		JP Peony Root		JP Glycyrrhiza				JP Glycyrrhiza	
			JP Moutan Bark	JP Astragalus Root	JP Powdered Processed Aconite Root		JP Ephedra Herb		JP Ginger					
			JP Cinnamon Bark	JP Schizonepeta Spike										
			JP Powdered Processed Aconite Root	JP Glycyrrhiza										

*JP, The Japanese Pharmacopoeia*.

Orengedokuto, tokiinshi, goshajinkigan, hachimijiogan, and rokumigan were recommended in patients with skin symptom. In the kampo concept, orengedokuto is used to improve inflammation in skin and other organs and improve irritation. Therefore, it should be used in patients with reddish skin and worsening itchy symptom with irritation. Goshajinkigan, hachimijiogan, and rokumigan were categorized in similar kampo medicine. They have been used for dry skin, urinary disorder, and sarcopenia. These kampo medicine include crude drugs to moisturize the skin, strengthen the muscles, promote microcirculation, and control urination. Additional effects include the following: for tokiinshi, suppressing itchiness, for goshajinkigan, warming the body and improving edema and lumber pain, for hachimijiogan, controlling cold and hot feeling and improving edema, and for rokumigan, controlling hot feeling and improving edema. These kampo medicines can be selected depending on not only the skin condition but also cold/hot sensation and water balance. Kishida et al. reported that goshajinkigan suppressed sarcopenia via the IGF-1/insulin pathway, maintained the expression of mitochondrial-related transcription factors, and suppressed TNF-α in SAMP8 mice, indicating that goshajinkigan is a promising candidate for relief from sarcopenia (41). CPG and results from the study suggested that goshajinkigan can be applied for skin symptom with additional expected effect on sarcopenia.

Shoseiryuto was selected for use in patients with allergic rhinitis and cough. Bakumondoto was selected for use in patients with cough. These kampo medicines have opposite effects on water imbalance in the upper respiratory tract. Shoseiryuto can improve the occurrence of excess water in thin secretion, thus it is applied in cases with allergic rhinitis and cough with thin secretion. Shoseiryuto decreased the number of T-helper (Th) 2 cells and the level of interleukin-4. The Th1 cells were not altered (42). Since shoseiryuto does not affect the histamine H1 receptors, there are fewer side-effects such as sedation and drowsiness. On the other hand, bakumondoto can moisturize and improve the dryness and sensitivity of the upper respiratory tract epithelium. Thus, it is applied in case of dry cough without secretion after respiratory infection. Ophiopogonis Radix, another herb in bakumondoto, has anti-inflammatory activity; and its active components include ruscogenin and ophiopogonin D.

Rikkunshito was recommended for patients with PPI-refractory GEARD and FD. It supplies vital energy to the digestive organs and promotes peristalsis from the stomach to the intestine. Thus, this kampo medicine is more suitable for patients with fatigue, anorexia, and gastric hypomotility resulting in weight loss. A recent study reported that rikkunshito increased plasma ghrelin levels in humans and mice (43) and restored the decreased plasma ghrelin levels induced by serotonin release in rats.

Keishikashakuyakuto was recommended for irritable bowel syndrome. It can be applied for both diarrhea, constipation, and mix-type IBS. It can control contraction and expansion of the intestine, resulting in pain relief and control of the stool condition.

Daiokanzoto and daikenchuto were recommended for constipation. Daiokanzoto is applied for constipation in patients whose vital energy is sufficient; whereas, daikenchuto is applied for constipation in patients with low vital energy, and characteristics of low body weight and abdominal distention, because it includes the crude drug, ginseng, which can supply energy to the digestive function. Daikenchuto treats abdominal symptoms via enhancing the secretions of motilin (44), substance P (45), calcitonin gene-related peptide, and adrenomedullin (46, 47), and activating the transient receptor potential of the vanilloid receptor complex (48).

Yokukansan was recommended for patients with BPSD. It has been used for irritation and sleep disorder of children since ancient times. Recently, it has been applied for the symptom in elderly patients. With regard to the characteristics of yokukansan, it does not induce extrapyramidal symptoms, however, it controls the concentration of serotonin in the synaptic cleft via partial agonist action on the serotonin 1A receptor (49), downregulates expression of the serotonin 2A receptor (50), inhibits the release of glutamate resulting in a decreased concentration of glutamate at synaptic clefts in the brain, and promotes glutamate uptake by astrocytes (51–53).

Hangekobokuto was recommended for use in patients with dysphagia. It has been used for pharyngeal discomfort since ancient times. It can soothe sensation in the pharynx and improve swallowing and cough reflex. It is reported that hangekobokuto modulates cerebral levels of 5-hydroxytryptamine, noradrenaline, and dopamine in mice (54).

There are some advantages in kampo treatment. As a super-aging society, the increased cost of medical insurance is a serious problem in Japan. The medical cost of kampo medicine is relatively lower than that of western medicine. This treatment can improve the target symptoms and contribute to holistic control of whole-body conditions, as mentioned above. Furthermore, kampo medicine has been applied for disease prevention and maintenance of quality of life since ancient times. Kampo medicine may contribute to health in elderly individuals. Based on these aspects, both CPG and research regarding kampo medicine for geriatric patients should be continued. The clinical application of kampo medicine has been performed depending on the patients' clinical history and physical sign. In this review, we demonstrated the present status of CPG involving kampo medicine for geriatrics. Clinical and basic research studies are currently on going. In future, more information on evidence-based detailed application will become available.

### Limitation

A limitation of this review is that the target subjects of the RCT referenced in the CPG do not necessarily correspond to elderly patients. The articles cited in ref. (16–19, 22, 23) were limited in their study of elderly subjects. However, the articles cited in ref. (20, 21, 25–33) included elderly subjects as a portion of the study population. The number of RCTs with only elderly subjects was very small; thus, we included studies with elderly subjects as a portion of the study population. Despite this limitation, this review demonstrated the use of Japanese CPG and kampo medicine in the treatment of symptoms in elderly patients.

## Conclusion

Japanese CPG gave some recommendation for use of kampo medicine for symptoms in the elderly. In the CPG, evidence related to kampo medicine for several symptoms is provided, and each kampo medicine includes multiple crude drugs that have multiple pharmacological functions. Thus, the treatment can be considered as holistic medicine which can lead to support of elderly patients with frailty.

## Author contributions

ST designed the study, confirmed the CPG and articles, and wrote the manuscript. SK proof read the CPG and the manuscript. AK, MO, RA, and TI gave advice for the study design and manuscript.

### Conflict of interest statement

AK, MO, ST, and TI belong the Department of Kampo and Integrative Medicine, Tohoku University School of Medicine. The department received a grant from Tsumura, a Japanese manufacturer of Kampo medicine; however, the grant was used as per Tohoku University rules. The remaining authors declare that the research was conducted in the absence of any commercial or financial relationships that could be construed as a potential conflict of interest.
